# Toxicity of Ochratoxin A and Its Modulation by Antioxidants: A Review

**DOI:** 10.3390/toxins5101742

**Published:** 2013-10-11

**Authors:** Valeria Sorrenti, Claudia Di Giacomo, Rosaria Acquaviva, Ignazio Barbagallo, Matteo Bognanno, Fabio Galvano

**Affiliations:** 1 Department of Drug Science, Section of Biochemistry, University of Catania, Catania95125, Italy; E-Mails: cdigiaco@unict.it (C.D.G.); racquavi@unict.it (R.A.); ignazio.barbagallo@unict.it (I.B.); fgalvano@unict.it (F.G.); 2 Agriculture Department, Mediterranean University of Reggio Calabria, Reggio Calabria89122, Italy; E-Mail: matteo.bognanno@unirc.it

**Keywords:** ochratoxin A, oxidative stress, DNA damage, antioxidants

## Abstract

Ochratoxin A (OTA) is a mycotoxin involved in the development of different types of cancers in rats, mice and humans. A growing number of *in vitro* and *in vivo* studies has been collected and has described evidence compatible with a role for oxidative stress in OTA toxicity and carcinogenicity. Because the contribution of the oxidative stress response in the development of cancers is well established, a role in OTA carcinogenicity is plausible. Several studies have been performed to try to counteract the adverse effects of oxygen radicals generated under OTA-exposure. A number of molecules with various antioxidant properties were tested, using *in vivo* or *in vitro* models. Protection against OTA-induced DNA damage, lipid peroxidation, as well as cytotoxicity were observed, further confirming the link between OTA toxicity and oxidative damage. These studies demonstrated that antioxidants are able to counteract the deleterious effects of chronic consumption or exposure to OTA and confirmed the potential effectiveness of dietary strategies to counteract OTA toxicity.

## 1. Overview of Mycotoxins

Mycotoxins are secondary metabolites of some fungi belonging to *Aspergillus*, *Penicillium* and *Fusarium* species and are common contaminants of human foodstuffs, such as wine, coffee beans, nuts and animal feed. Mycotoxins can enter the food chain through contaminated cereals and foodstuffs (e.g., milk, meat, eggs) obtained from animals fed mycotoxin-contaminated feedstuffs. Mycotoxins can cause serious health problems in animals and humans known as mycotoxicosis. The major problem associated with animal feed contaminated with mycotoxins is not acute disease episodes, but rather, the ingestion of low levels of toxins, which may cause an array of metabolic, physiologic and immunologic disturbances. The most extensively investigated mycotoxins are aflatoxins (AF), deoxynivalenol (DON), zearalenone (ZEN), fumonisin B1 (FB1) ochratoxin A (OTA) and T2 toxin [1,2,3,4,5].

Mycotoxins are challenging to classify, due to their diverse chemical structures and biosynthetic origins, their myriad of biological effects and their production by a wide number of different fungal species. Thus, mycotoxins can be classified as hepatotoxins, nephrotoxins, neurotoxins, immunotoxins, teratogens, mutagens, carcinogens, allergens, and so forth. Moreover, by their chemical structures, mycotoxins can be classified as lactones and coumarins, according to their biosynthetic origins, as polyketides, amino acid-derived, *etc*., and, finally, by the fungi that produce them (e.g., *Aspergillus* toxins, *Penicillium* toxins). Then, the same compound may get placed in different cognitive cubbyholes. Aflatoxin, for example, is a hepatotoxic, mutagenic, carcinogenic, difuran-containing, polyketide-derived *Aspergillus* toxin. Zearalenone is a *Fusarium* metabolite with potent estrogenic activity. 

Some mycotoxins are specifically indicated or strongly suspected as the cause of severe human and animal diseases, such as Reye’s disease, caused by aflatoxin B1 (AFB1), equine leukoencephalomalacia and porcine pulmonary edema, caused by fuminisin B1 (FB1), human alimentary toxic aleukia, caused by T2 toxin, and Balkan endemic nephropathy, caused by ochratoxin A. The positive correlation between the consumption of AFB1-contaminated foods and the increased incidence of liver cancer in several Asian and African populations led to the classification of AFB1 as a group 1A carcinogens by the International Agency for Research on Cancer [6].

The first Joint Food and Agricultural Organization (FAO)/World Health Organization (WHO) Conference on Food Additives, was held in September, 1955 [7], and since then, there have been 76 meetings of the Committee. In 1991 the joint FAO/WHO Expert Committee on Food Additives (JECFA) first evaluated OTA and, based on the LOAEL (Lowest Observed Adverse Effect Level) in pig, allocated a Provisional Tolerable Weekly Intake (PTWI) of 112 ng/kg body weight (bw) [8]. This value was rounded to 100 ng/kg bw/week and confirmed in several subsequent meetings [9,10].

In 1997, the Joint FAO/WHO Expert Committee on Food Additives provided qualitative and quantitative information on aflatoxins and concluded that aflatoxins should be treated as carcinogenic food contaminants, the intake of which should be reduced to levels as low as reasonably achievable [11]. In 2002, the Joint FAO/WHO Expert Committee on Food additive evidenced that several mycotoxins may exist in many contaminated foodstuffs and foodstuff products. Moreover, contaminated feeds are frequently more toxic than the pure toxin in animals and humans, indicating possible interactions. The Committee, therefore, recommended further studies on mycotoxins occurring concomitantly in foods, their possible interactions and how the toxicological significance of such interactions could be assessed [10].

In 2007, the Joint FAO/WHO Expert Committee on Food Additives noted that the reduction of dietary AF exposure is an important public health goal, particularly in populations that consume high levels of any potentially AF-contaminated food. Moreover the Committee reported new data on estimates of dietary exposure to OTA, which causes various toxic effects, such as neurotoxicity, immunotoxicity, nephrotoxicity and genotoxicity [12]. In 2008, JECFA applied a benchmark dose (BMD) modeling approach using carcinogenicity data [13].

Most studies were conducted *in vitro* and *in vivo* on mycotoxins, particularly on AFB1, OTA and FB1. This review focuses on ochratoxin A toxicity and the protective effects of antioxidants.

## 2. Ochratoxin A

OTA, or (*R*)-*N*-[(5-chloro-3,4-dihydro-8-hydroxy-3-methyl-1-oxo-1*H*-2-benzopyran-7-yl)carbonyl)-l-phenylalanine, is a naturally occurring mycotoxin soluble in organic solvents, in aqueous solution of sodium bicarbonate and slightly soluble in water. OTA is efficiently absorbed from the gastrointestinal tract, mainly in the small intestine. Information from a number of species shows that it is distributed via the blood, mainly to the kidneys, with lower concentrations found in liver, muscle and fat. Specific transporters may be involved in the cellular uptake of ochratoxin A into the kidney, where it accumulates [14]. Transfer to milk has been demonstrated in rats, rabbits and humans, but little OTA is transferred to the milk of ruminants, owing to efficient hydrolysis of the amide bond by microflora in the rumen, to yield phenylalanine and ochratoxin *alpha*. Ochratoxin *alpha*, a chlorinated dihydroisocoumarin, is not the only metabolite of OTA. There are several papers dealing with biotransformation of OTA into several metabolites, such as Ochratoxin B (OTB), open-ring ochratoxin A (OP-OA), 4 hydroxylated OTA, 10 hydroxylated OTA, OTA without phenylalanine, OTB without phenylalanine, OTA hydroquinone (OTHQ) and a dechlorinated ochratoxin A derivative. Some of these, such as OTHQ and OP-OA, are much more toxic than OTA itself [15,16,17,18]. OTA is produced by several fungi of *Aspergillus* and *Penicillium* species, often found in a variety of food commodities, such as cereals, green coffee, cocoa, dried fruits and meat products, resulting in continuous exposure of the human population to OTA [19]. OTA has been shown to be nephrotoxic, hepatotoxic, teratogenic and immunotoxic to several species of animals and is known to cause kidney and liver tumors in mice and rats [20,21,22]. In humans, it has been related to Balkan endemic nephropathy [23,24,25,26], although this hypothesis has not been completely demonstrated. Recently, other nephrotoxic agents have been put forward as the primary cause of Balkan endemic nephropathy (BEN) [27,28,29].

In farm animals, chronic exposure to OTA produces a well-described nephropathy [30]. OTA is a mycotoxin, with important implications for animal and human health. Exposure to OTA is a worldwide phenomenon, as evidenced by its detection in sera from human individuals of many countries.

However, human epidemiology has been inconclusive. Epidemiological data were recently reviewed by several expert groups [31,32,33]. All concluded that the causality between the intake of OTA and human nephropathy could not be established. Therefore, the International Agency for Research on Cancer (IARC) statement that there is inadequate evidence for carcinogenicity in humans (group 2B) appears still valid [6].

In absence of adequate human data, risk assessments have relied on studies conducted in laboratory animals, where it causes various toxic effects, the most relevant being nephrotoxicity and nephrocarcinogenicity in rats [33]. A number of mechanisms have been proposed to account for OTA toxicity and OTA-induced renal tumor formation: inhibition of protein synthesis, interference with metabolic systems involving phenylalanine, promotion of membrane lipid peroxidation, disruption of calcium homeostasis, inhibition of mitochondrial respiration and DNA damage [15,34,35,36,37,38]. Other research has focused on the ability of OTA to disturb cellular signaling and regulation, as well as to modulate physiological signals, known to influence cell viability and proliferation. Recent studies have specifically focused on (i) metabolism-mediated toxicity via oxidative stress, (ii) intracellular OTA accumulation as a function of organic anion transporters and (iii) inter- and intra-cellular signal transduction at nanomolar concentrations [39,40].

In a review by O’Brien and Dietrich [41] on its toxicological properties, OTA has justly been called “the continuing enigma”. According to the authors, it is not yet clear whether the predominant toxic mechanism of OTA is of a genotoxic or epigenetic nature, such as induced cytotoxicity, oxidative cell stress or increased cell proliferation, due to an imbalance between proliferative and antiproliferative intracellular signal pathways. However, both the carcinogenicity and cytotoxicity of OTA have been related to free radical-mediated oxidative cell damage [42,43,44,45,46,47,48,49,50,51]. Schaaf *et al*. [43] attributed proximal tubule cell damage caused by OTA to the formation of reactive oxygen species, such as the superoxide anion (O_2_∙), hydroxyl radical (∙OH) and peroxide (ROO∙), which induce a wide range of lesions in cell components. Other authors reported that the hydroxyl radical was not involved in the process, but a role for cytochrome P450 in this reaction was suggested: CYP450 is able to stimulate OTA-dependent lipid peroxidation and this action is mediated by OTA-Fe^3+^/^2+^ complexes [52,53,54]. It has been shown that oxidative damage contributes to the wide range of toxic effects of OTA [47,55,56].

Structure-activity studies have also suggested that the toxicity of OTA may be attributable to its isocoumarin moiety and that the lactone carbonyl group may be involved in its toxicity. Using a *Bacillus brevis* model, Hoehler’s *et al*. showed that OTA behaved as a cell pro-oxidant through mobilization of the Fe^2+^ and Ca^2+^ pathways, leading to uncoupling oxidative phosphorylation and increased production of hydroxyl radical via the Fenton reaction [45]. However, in other studies using OTA and structural analogs, a direct correlation between toxicity and iron chelating capacity was only partially supported [57]. The generation of an OTA hydroquinone/quinone couple from the oxidation of OTA (phenol oxidation) by electrochemical, photochemical and chemical processes was reported [17,18,44,58]. Quinone is thought to undergo reductions to form hydroquinone, postulated to be responsible for the formation of the glutathione conjugate of OTA. Such events are likely to result in redox cycling and in the generation of reactive oxygen species [16,44,59]. The formation of OTA-derived quinones has been observed in cell cultures *in vitro* [16] as well as *in vivo* [26,60].

Our previous study [61] was aimed at verifying whether OTA is related to free radical-mediated oxidative cell damage. Male Sprague-Dawley rats received the control diet supplemented with 200 parts per billion of OTA. After four weeks of treatment, animals were killed, and the liver, kidneys and brain of each rat were collected and homogenized to evaluate non-proteic thiol groups (RSH), lipid hydroperoxide (LOOH) levels and DNA fragmentation. We observed that OTA induced alterations in LOOH and RSH levels, confirming the involvement of the oxidative pathway in damage induced by OTA in all the examined tissues compared with the control group. Analysis of DNA fragmentation evidenced that, following chronic consumption of OTA, DNA damage occurred in all three tissues under examination. These results confirm that kidney is the target organ, but also demonstrate that OTA toxicity to other organs should not be underestimated. Moreover, Cavin *et al*. [34] reported an OTA-mediated increase of the inducible nitric oxide synthase (iNOS) expression in a normal rat kidney cell line and in rat hepatocyte cultures, suggesting the induction of both oxidative and nitrosative stress. Strong evidence suggests that nitric oxide (NO), produced by three isoforms of nitric oxide synthases (neuronal NOS, endothelial NOS and inducible NOS), mediates a variety of actions, such as vasodilatation, neurotransmission, host defense against bacteria and angiogenesis [62,63].

Although conflicting data has been reported, an overwhelming amount of clinical and experimental evidence suggests a positive association between iNOS/endothelial NOS (eNOS) overexpression, NO production and tumor progression [64,65,66,67]. In particular, NO produced by eNOS may be involved in tumor angiogenesis [68]. Modulation of NO production may therefore play an important role in the regulation of angiogenesis and, consequently, in tumor progression. Kostorou *et al*. reported the involvement of dimethylarginine dimethylaminohydrolase (DDAH) in cerebral tumor growth and the development of tumor vasculature [69]; this enzyme metabolizes the endogenous NOS inhibitor, asymmetric dimethylarginine (ADMA). Two isoforms of DDAH with distinct tissue distribution have been identified: DDAH-1 and DDAH-2 [70]. Both isoforms have been identified in the kidney and liver tissues, but the expression of the DDAH-1 isoform appears more abundant [71,72]. In consideration of OTA nephrotoxicity and its possible involvement in the development of urinary tract tumors and, also, in view of the involvement of DDAH and NOS in tumor growth and the development of tumor vasculature, the aim of our subsequent study was to evaluate the effect of chronic OTA-exposure on the DDAH/NOS pathway in rats [73]. The experiments were performed in male Sprague-Dawley albino rats treated under the same experimental conditions of our previous study [61]. After four weeks of daily treatment, liver and kidneys were processed for eNOS, iNOS and DDAH-1 Western blotting, nitrite level evaluation and DDAH activity determination. It has been reported that the kidney is the target organ of OTA toxicity, probably because OTA is actively accumulated in kidney cells [74]. Nevertheless, OTA has been shown to affect other organs, as well, including the liver. OTA has been shown to be hepatotoxic in rats [75]. Recently, OTA renal and hepatic carcinogenicity was also observed in chicks [76].

Although the liver is not the main target organ for OTA, hepatocytes are exposed to OTA, since it has to pass through the liver after intestinal absorption [77]. Results obtained in our study, in line with *in vitro* studies by Cavin [34] and Ferrante [78], allow us to suggest that, through iNOS induction, OTA is able to induce overproduction of NO, both in kidney and liver, resulting in increased nitrite and nitrate levels. Under normal conditions, NO presents a broad range of biological activities; conversely, in excess, it may behave as a toxic radical. In fact, NO is known to react with O_2_∙ to form the pro-oxidant peroxynitrite, ONOO^−^ [79], with consequent nitrosative stress. As reported in our previous research [61], four-week OTA exposure is able to induce oxidative damage, both in kidney and in liver; then, overproduction of OTA-induced NO, in the same experimental conditions, may form the pro-oxidant ONOO^−^, both in kidney and liver. However, our data demonstrate that, only in kidney, OTA is also able to induce eNOS and DDAH-1 overexpression and DDAH activation with a further increase of NO levels. These data allowed us to speculate that, even if four weeks of exposure to OTA were much too low to induce renal tumors, one of the many possible mechanisms by which long-term OTA exposure may cause nephrocarcinogenity might consist in eNOS-DDAH involvement. Therefore, modulation of NO production may play an important role in regulation of angiogenesis and, consequently, in tumor progression.

### 2.1. Is OTA a Genotoxic or Non-Genotoxic Carcinogen?

Over the last few decades, studies aimed at elucidating the modes of action implicated in OTA toxicity and carcinogenicity have been published [80]. There has been considerable debate for many years over the genotoxicity of OTA and its actual role in carcinogenicity [8,9,10,13,31,32,33,81,82,83]. Several authors and expert groups have concluded that OTA is genotoxic [35,37,38]. However, other authors indicate that OTA is unlikely to act through a direct genotoxic mechanism [12,32] and that its carcinogenicity is due to an indirect mechanism, such as induction of oxidative stress [36,87].

The genotoxic and mutagenic activity of OTA has been assessed in a variety of standard tests, in order to evidence direct DNA damage that could be the origin of the mechanism involved in chemical carcinogenesis, but the results that have been published are controversial. OTA was originally regarded as a non-mutagenic compound, because most bacterial assays gave negative results [85,86,87,88,89]. Positive results using *Salmonella typhimurium* Ames strains have only been reported using OTA-exposed hepatocyte culture medium [90] or in the presence of kidney microsomal fractions, instead of hepatic ones, as the metabolic activation system [91]. These results suggested an important role on the part of metabolism in the genotoxicity of OTA and/or a selective toxicity in target kidney cells. In assays that detect unscheduled DNA synthesis due to repair processes, contradictory results have also been found. Bendele *et al*. [87] did not detect DNA repair synthesis induction in primary rat hepatocyte cultures; Mori *et al*. [92] found a weak DNA repair synthesis induction in mice and rat hepatocytes; a more pronounced effect was found in porcine urinary bladder epithelial cells by Dörrenhaus and Föllmann [93]. These results pointed to a selective toxic effect of OTA in target cells.

The potential of OTA to form covalent DNA adducts has been subjected to debate, due to conflicting data in the literature. Using ^32^P-postlabelling analysis, large numbers of OTA-derived DNA adducts have been reported to be present in a wide range of tissues from OTA-treated rats, mice, as well as pigs [15,59,82,94,95,96]. However, according to some authors, these adducts have never been observed by any other highly-specific techniques, such as radioactivity measurements using ^3^H-labelled OTA (^3^H-OTA) accelerator mass spectrometry (AMS) or liquid chromatography-tandem mass spectrometry (LCMS/MS) [97,98], while for others, the formation of DNA adducts has been proven by isotope dilution LC-MS/MS [95]. While results reported by Schlatter *et al*. and by Delatour *et al*. [97,98] indicate that covalent binding of OTA or some of its metabolites to DNA is not produced, therefore excluding the possibility of a genotoxic mechanism in the carcinogenicity of OTA, results reported by Mantle *et al*. [95] would not exclude it.

As reported by Pfohl-Leszkowicz A. [99], the ^32^P-postlabelling method evidences both adducts that contain the OTA itself and adducts that contain OTA metabolites, such as quinone (OTQ), derived as the products of oxidative stress. Nevertheless, other data suggest the idea that the phenoxyl radical of OTA can have a role in DNA adduction *in vivo* [16].

In the different assays that measure DNA fragmentation and chromosome aberrations, OTA has always given positive results. It has been shown to cause single-strand DNA breaks in rat and mice kidney and liver [100,101,102] and also in target and non-target cell lines of different species [35,56,103,104]. Many studies have been performed with the comet assay, and a few have evaluated oxidative DNA damage [56,100]. It has also been found that OTA induces micronuclei in cell lines of different origins, Hep G2, SHE (Syrian Hamster embryo) and OSV (ovine seminal vesicle) [103,105,106]. With the aim to study the ability of OTA to cause DNA damage in human kidney cells derived from proximal tubular cells, which are the toxic target, Arbillaga *et al*. [107] assayed the ability of OTA to induce DNA strand breaks and oxidative DNA damage using the alkaline comet assay in the human renal proximal tubular epithelial cell line (HK-2). Obtained data suggest that OTA is not acting as a direct genotoxic carcinogen and that oxidative stress is implicated in the genotoxicity and cytotoxicity observed in these human renal cells. In the same cell line (HK2), as well as in rat liver and kidney, covalent DNA adducts have been observed and related to OTA biotransformation [17,108]. Although, oral exposure to mycotoxins is the common route, it has been suggested by WHO that because of the employment of manual labor during the pre- and post-harvest stages of agriculture, dermal exposure to these chemicals may also occur [109]. Therefore, Abel *et al*. [110] employed a two-stage mouse skin tumorigenesis model to study the tumorigenic property of OTA and related molecular events. Recently, Kumar *et al*. reported that topical application of OTA causes DNA damage and tumor initiation in mouse skin [111]. It has been well established that DNA damage is an important event in the initiation of chemical carcinogenesis [112]. Although conflicting data are reported regarding evidence of OTA-DNA adduct formation [16,17,51,60,113,114,115,116], the DNA damaging potential of OTA has been reported by various investigators, both *in vivo* and *in vitro* [32,41,56,83,100]. Therefore, it has been suggested that ROS and oxidative DNA damage could be one of the causative factors for OTA-induced toxicity and tumorigenicity [117]. Kumar *et al*. [111] revealed that OTA has a skin tumor initiating property under *in vivo* conditions, which may be related to oxidative stress, Mitogen-activated protein kinases (MAPKs) signaling and DNA damage in mouse skin, and antioxidants may have a role in the prevention of OTA-induced tumorigenesis, which needs to be investigated.

OTA has been found to induce an increase in ROS levels and oxidative damage in some immortalized renal cell and cancer cell lines, such as human renal proximal tubular epithelial cells (HK-2), primary rat proximal tubular cells, proximal tubular cells (LLC-PK1), human hepatoma-derived cells (HepG2) and human colonic adenocarcinoma cells (CaCo-2) [43,107,118,119]. Cui *et al*. showed that OTA could induce G2 phase arrest and apoptosis in immortalized human gastric epithelial cells (GES-1) [120]. Though most of the studies on OTA have been focused on renal toxicity or carcinogenicity [15,121], the immunosuppressive effects of OTA caused more and more attentions in the biomedical field. It is worth noting that OTA is frequently found in human blood, due to its widespread contamination in food and grains [24,122,123]. More importantly, OTA is a persistent toxin, which, following a single oral dose, remains in human circulation for a long time, due to the unfavorable kinetics of renal elimination [97,124]. As reported by Castegnaro *et al*. [125], with an average weekly intake of OTA that varies from 1.9 to 206 ng/kg body weight (twice the tolerable weekly intake recommended by JECFA), OTA blood concentrations are in a range reaching 10 µg/L.

Thus, it is quite reasonable for the objective evaluation of the hazardous bioeffects of OTA exposure on human to explore the possible effects of OTA on the immune cells in human peripheral blood. Assaf *et al*. showed that OTA could induce immunosuppression via marked apoptosis in human lymphocytes [126]. It has been generally accepted that the induction of cell cycle arrest and apoptosis was an important bioeffect of many carcinogenic mycotoxins.

Liu *et al*. hypothesized that OTA exposure in blood might induce cell cycle arrest and apoptosis also in human peripheral blood mononuclear cells (hPBMC). The authors reported that in hPBMC, oxidative stress is involved in OTA-induced human immunotoxicity, and they conclude that OTA-induced DNA damage and G1 arrest may play roles in the carcinogenesis of OTA [127].

Moreover, studies have shown that OTA is produced by phytopathogenic *Aspergillus ochraceus* and *Aspergillus carbonarius* strains, suggesting that this toxin may play a role in the etiology of plant diseases [128]. The plant response to attempted infection by microbial pathogens is often accompanied by rapid cell death in and around the initial infection site, a reaction known as the hypersensitive response (HR). Xenobiotics could also induce HR-like lesions. In the presence of OTA, the growth of *Arabidopsis thaliana* on media was significantly inhibited; in addition, cell death was observed with features resembling the HR-type lesions in excised leaves that were infiltrated with this toxin. There was also evidence that cell death was induced by OTA, such as the occurrence of an oxidative burst and the deposition of callose and phenolic compounds (autofluorescence) [129].

## 3. Ochratoxin A Toxicity: The Effect of Antioxidant Substances and Food Components

Due to its widespread threat to human health, the detoxification of OTA has been of major interest to researchers. Physical, chemical and biological methods have been developed to reduce and/or eliminate the toxic effects of contaminated products, improve food safety and minimize economic losses [130]. However, the process of detoxification is often accompanied with a loss of palatability and nutritional values. The addition of nutrients or additives with protective properties to contaminated foodstuffs is one approach that reduces the toxicity of mycotoxins.

A growing number of *in vitro* and *in vivo* studies has been collected and described evidence compatible with a role for oxidative stress in OTA toxicity and carcinogenicity. For these reasons, several studies have been performed using antioxidants to try to counteract the adverse effects of oxygen radicals generated under OTA-treatment. Some of these studies are elaborately discussed below.

### 3.1. Vitamins

α-Tocopherol is a member of the vitamin E compound group that has several biological roles [131,132]. Vitamin E is a potent antioxidant; its function as a peroxyl radical scavenger that terminates chain reactions is well documented [133,134]. In one human and four animal cell lines, Baldi *et al*. [135] determined the half lethal concentration (LC_50_) of OTA, its effect on ROS production, and its ability to induce cytochrome P_450_ activity. They also examined the protective effect of α-tocopherol in the most sensitive cell lines (bovine mammary epithelial cells (BME-UV1) and Madin Darby canine kidney cells (MDCK)). Pre-incubation for 3 h with α-tocopherol significantly ameliorated the OTA-induced reduction in cell viability and significantly decreased ROS production. These findings indicate that oxidative stress is an important factor in OTA cytotoxicity, and supplementation with α-tocopherol may counteract OTA cytotoxicity. Fusi *et al*. [38] studied the role of α-tocopherol in counteracting several types of damage induced by OTA in primary porcine fibroblast cultures. OTA cytotoxicity developed through several mechanisms of action, such as Lactate dehydrogenase (LDH) release and DNA fragmentation. α-Tocopherol treatments reduce the damage induced by OTA at different cellular levels. The authors concluded that the use of α-tocopherol could offer new strategies to reduce OTA cytotoxicity, supporting its defensive role in the cell membrane and its multiple functions in cellular metabolism. Grosse *et al*. [50] reported the effects of some vitamins, such as retinol (A), ascorbic acid (C) and α-tocopherol (E), which are known to act as superoxide anion scavengers, on OTA genotoxicity. Pretreatment of mice by vitamin E decreased DNA adducts by 80% in kidney. Vitamin A decreased DNA adduct levels by 70% and vitamin C, by 90% in kidney. The decrease of the genotoxicity of OTA by vitamin A is due to the antioxidant properties of vitamin A. The decrease of the genotoxicity of OTA by vitamin E is due to the scavenging of lipid hydroperoxyl radicals by vitamin E. This is due to the increase of glutathione peroxidase, which utilizes glutathione (GSH) to catalyze the reduction of hydroperoxides. Vitamin E also induced Nicotinamide adenine dinucleotide phosphate reduced (NADPH):quinone reductase and glutathione S-transferase, which are detoxifying enzymes that reduce quinones. Ascorbic acid is the most efficient vitamin for inhibiting OTA genotoxicity. Vitamin C is an important antioxidant and a free radical scavenger, thereby preventing the production of electrophilic metabolites. Moreover, it is indispensable for the regeneration of vitamin E in lipid membranes and acts synergistically with other biological antioxidants, such as glutathione. This vitamin also decreased the activities of several cytochrome P_450_ isoenzymes.

### 3.2. Phenolic Compounds

Catechins are a class of phenolic compounds presents in green tea leaves, chocolate and some plants. They have been shown to have several healthy properties. Among them are anticancer properties [136,137] and protective capacity against oxidative stress-related diseases [138,139,140,141]. Costa *et al*. investigated the protective effect of two catechins (epigallocatechin gallate (EGCG) and epicatechin gallate (ECG)) against OTA-induced cytotoxicity in a pig kidney cell line (LLC-PK1) [142]. The ability of the catechins to reduce ROS production and DNA fragmentation induced by OTA was also investigated. Costa *et al*. reported the cytoprotective effects of catechins *in vitro* from OTA-induced cell damage and a good scavenging power based on the inhibition of ROS production. In particular, a 24-h pre-treatment with EGCG or ECG restored cell viability with respect to OTA alone. Pretreatment with EGCG at low concentration for eight days protected cells from OTA-induced cell death. Moreover, both catechins reduced OTA-induced ROS production. A reduction of OTA-induced DNA fragmentation was found for LLC-PK1 cells pre-treated with EGCG and ECG. The free-radical scavenging capacity of both catechins was tested with the Briggs-Rauscher oscillating method and the Trolox equivalent antioxidant capacity (TEAC) assay. The results show a good scavenging power in accordance with the inhibition of ROS production. The authors concluded that catechins could be useful for developing alimentary strategies for both humans and animals to prevent OTA-induced cytotoxicity. Corcuera *et al*. prepared a polyphenol-enriched cocoa extract (PECE) and evaluated its ability to reduce OTA cytotoxicity and ROS induction in a cell-free system and in Hep G2 cells. Results reported by Corcuera *et al*. evidenced that polyphenols extracted from cocoa have a good antioxidant activity and may be efficient at reducing the generation of ROS produced by mycotoxins or other oxidant agents [143]. The aim of our previous study [61] was to verify whether the oral administration of cyanidin 3-O-β-D-glucoside (C3G), an anthocyanin largely present in the human diet through beans, fruits, vegetables and red wine, might counteract damage induced by chronic exposure to OTA in rats and if its effect may be mediated by heme oxygenase-1 (HO-1). Male Sprague-Dawley rats were divided into four groups of ten animals. A control group received a commercial diet. Group C3G received the control diet supplemented with C3G (1 g/kg feed). Group OTA received the control diet supplemented with 200 parts per billion of OTA, and group OTA *plus* C3G received the OTA group diet supplemented with C3G. After four weeks of treatment, animals were killed, and the liver, kidneys and brain of each rat were processed as described above. In the OTA *plus* C3G group, both RSH content and LOOH levels were similar to those observed in the control group, demonstrating that C3G was able to counteract the effects of OTA. A significant induction of HO-1 was evident in kidney and liver of both OTA and C3G groups. DNA damage occurred in all the examined tissues of the OTA group, whereas C3G was able to prevent it. Results obtained in this study confirmed that the effects of OTA are mediated by oxidative stress and demonstrated that C3G efficiently counteracted the deleterious effects of OTA because of its antioxidant and HO-1-inducing properties. Since it has been reported that naturally occurring antioxidants that potently induce HO-1 expression lead to an increased resistance to oxidative stress-mediated damage, the beneficial actions attributed to several natural substances, such as C3G, could be due to their intrinsic ability to activate the HO-1 pathway [144,145,146].

Moreover, another of our previous studies [73] demonstrates that C3G, besides its well-known antioxidant activity, may also act with different molecular mechanisms. For this study, male Sprague-Dawley rats were treated and liver and kidneys were processed as described above. eNOS, iNOS and DDAH-1 Western blotting were performed. Nitrite levels were evaluated, and DDAH activity was determined. It has been reported that in tumor growth and tumor angiogenesis are involved different enzymes, such as iNOS, eNOS and DDAH-1. We reported that in kidney of rats treated with OTA *plus* C3G, iNOS, eNOS and DDAH-1 expression levels were less pronounced compared with those observed in the OTA group. Coherent with decreased iNOS, eNOS and DDAH-1 expression, a decrease of nitrite and nitrate levels and of DDAH activity was observed in the OTA *plus* C3G group. These results allow us to speculate that long-term consumption of C3G might contribute toward reducing OTA-induced tumor growth and tumor angiogenesis in kidney.

Liquorice extract from the dried roots of *Glycyrrhiza glabra* L. (Fabaceae) is one of the herbal medicines that is used widely in various countries [147]. Extracted liquorice containing glycyrrhizin has been used as an additive for flavoring and sweetening tobacco, candies and beverages in many countries [148,149]. Vaya *et al*. demonstrated that liquorice plant extract (LPE) exerts a potent antioxidant capacity, as it possesses compounds, such as flavonoids [150]. Malekinejad *et al*. showed a protective effect of LPE on OTA-induced nephrotoxicity in rats [40]. Moreover, the authors in another study reported that histopathological analyses demonstrated that in OTA-exposed rats, testicular degeneration, seminiferous tubule atrophy, dissociation of germinative epithelium, vasodilatation with vascular thrombosis, perivascular immune cell infiltration, hypertrophied leydic cells, giant cell formation and a negative tubular differentiation index (TDI) were observed. They tested the effect of Glycyrrhiza glabra extract (GgE), as a natural antioxidant, and melatonin (MEL). Both the biochemical and histopathological examinations showed that MEL and GgE, albeit with some differences, exerted a protective effect on OTA-induced damages. The authors suggest that OTA contamination in animal feeds and human foods could cause reproductive abnormalities. Their data indicate that OTA, at least partly by interfering in the oxidative stress system, exerts its toxic effects on testes, whereas MEL and GgE, with antioxidant properties, could fairly protect rats against OTA toxic effects [151].

### 3.3. *Vitis* *vinifera*

Oral administration of OTA to young weanling mice (*Mus musculus*) caused several hematological changes and induced hepatoma and renal carcinoma. Concurrent administration of berry and leaf juice of the common grape (*Vitis vinifera*) to mice together with OTA significantly reduced the hepatic and renal damage caused by ingestion of this mycotoxin. None of the animals receiving berry/leaf juice of *Vitis vinifera* showed the formation of hepatorenal carcinoma, whereas 25% of animals receiving only OTA developed well-differentiated renal carcinoma and hepatic lesions [152].

### 3.4. Lycopene

Lycopene, the most prevalent carotenoid in the Western diet, is majorly present in tomatoes. The consumption of tomatoes and/or tomato products is associated with increased lycopene blood levels and reduced oxidative damage of lipids, proteins and DNA [153]. Lycopene has been suggested to have strong antioxidant potency *in vitro*, almost being 100 times more efficient in quenching singlet oxygen (^1^O_2_) than vitamin E [154]. Lycopene may act as a chemopreventive agent against certain types of cancers (i.e., cancers of prostate, stomach, breast and lung) and was found to be protective against chemotherapeutic-induced renal damage in several studies [155,156,157,158]. Palabiyik *et al*. [159] investigated the possible protective effect of lycopene against the renal toxic effects of OTA. Male Sprague-Dawley rats were treated with OTA (0.5 mg/kg/day) and/or lycopene (5 mg/kg/day) by gavage for 14 days. Histopathological examinations were performed, and apoptotic cell death in both cortex and medulla was evaluated by Terminal deoxynucleotidyl transferase dUTP nick end labeling (TUNEL) assay. OTA treatment was found to induce oxidative stress in rat kidney, as evidenced by marked decreases in catalase (CAT) activity and total glutathione (GSH) levels, as well as an increase in superoxide dismutase (SOD) activity. Furthermore, TUNEL analysis revealed a significant increase in the number of TUNEL-positive cells in cortex and medulla in the OTA-administered group compared to the control. Lycopene supplementation with OTA increased glutathione peroxidase (GPx) activity and GSH levels and decreased apoptotic cell death in both cortex and medulla *vs*. control. The results of this study showed that lycopene might be partially protective against OTA-induced nephrotoxicity and oxidative stress in rat, as evidenced by partial recovery in histopathology, apoptosis and antioxidant parameters. These data are in line with those of Baudrimont *et al*. [49].

### 3.5. Glutathione

GSH is a biological antioxidant and the major non-protein sulfhydryl present in cells; while its conjugation with strong electrophiles is considered a mechanism of cellular protection, certain conjugates act as toxicants in tissues rich in *γ*-glutamyl transpeptidase (*γ*-GT) [58]. A dual activity of glutathione with OTA has been reported: in liver, glutathione protects against genotoxicity, but increases the toxic effect in kidney. In fact, as reported by El Adlouni *et al*. [160], OTA is genotoxic and can be metabolized not only by different cytochrome P_450_ (CYP), but also by peroxidases involved in the arachidonic cascade. In kidney microsomes from rabbit, OTA biotransformation increased DNA-adduct formation through pathways involving microsomal glutathione-S-transferase and CYP2C9. Moreover, Faucet-Marquis *et al*. [16] reported that pretreatment of opossum kidney cells (OK) by modulators of glutathione pathways, such as 2-mercaptoethane sulfonate (MESNA) or N-acetylcysteine (another agent that, like MESNA, reduces oxidative stress by increasing free thiols in kidney), buthionine sulfoximine (BSO) (an inhibitor of glutathione-synthase) and alpha amino-3-chloro-4,5-dihydro-5-isoxazole acetic acid (ACIVICIN) (an inhibitor of gamma glutamyl transpeptidase), did not diminish OTA cytotoxicity significantly; indeed, ACIVICIN increased OTA cytotoxicity.

Toznolovanu *et al*. [108] reported that OTA, as well as electrophiles generated from its metabolism react with reduced glutathione (GSH) to produce GSH-conjugates. In kidney, OTB-GSH is the major metabolite. This was not the case in liver, where appreciable quantities of OTAα and OTHQ-GSH in addition to OTB-GSH were generated. These results provide insight to the susceptibility of rat kidney to OTA carcinogenesis. In kidney, higher levels of GSH conjugates, suggest a greater level of OTA bioactivation, which is required for DNA adduction. Moreover, in kidney, GSH conjugates may be involved in the nephrotoxicity, genotoxicity and carcinogenicity of OTA, as a consequence of the relatively high activity of *γ*-GT and dipeptidases within the brush border membrane of renal proximal tubular epithelial cells, whereas much higher levels of OTAα are detected in liver compared to kidney, and the formation of OTAα is a detoxification pathway for OTA, suggesting greater sensitivity of the kidney to OTA.

### 3.6. Zinc

Zinc is widely considered as a potential antioxidant *in vitro*. Zinc protects protein sulfhydryl groups against oxidation and decreases the formation of hydroxyl radical through the antagonism of redox-active transition metals, such as iron and copper [161]. Moreover, zinc is an essential component of Cu/Zn superoxide dismutase (SOD1). Zinc also regulates the expression of many genes that are involved in antioxidant processes, such as metallothionein (MT), GPx and glutamylcysteine synthetase (GCS), through the activation of metal response transcription factor-1 (MTF-1) [162]. Zheng *et al*. investigated whether zinc supplement could inhibit OTA-induced oxidative damage and DNA damage in HepG2 cells and the mechanism of inhibition [163]. They demonstrated that OTA toxicity is associated with the inhibition of cell proliferation, a decrease in the intracellular zinc concentration, the induction of ROS production, decreases in SOD activity and mitochondrial membrane potential (Δψm), DNA strand breaks, DNA oxidation and hypomethylation and 8-hydroxy-2′-deoxyguanosine (8-OHdG) formation. Moreover, zinc depletion by the zinc chelator, *N,N,N′,N′*-tetrakis (2-pyridylmethyl) ethylenediamine (TPEN), aggravates OTA-induced oxidative damage. Zinc supplement significantly reduced the OTA-induced production of ROS and a decrease in superoxide dismutase (SOD) activity; the protective effects of zinc against OTA toxicity may be related to its antioxidant properties and its involvement in redox signaling through regulating the expression of antioxidant proteins, such as MT, through the activation of MTF-1. Zinc also helps maintain the stability and integrity of DNA in OTA-treated cells by reducing DNA strand breaks and the formation of 8-OHdG and by reinstating DNA methylation. In the paper of Zheng *et al*., it was proven for the first time that zinc is able to reduce the cytotoxicity of OTA through inhibition of oxidative damage and DNA damage and regulation of the expression of zinc-associated genes. Thus, the addition of zinc can potentially be used to reduce the OTA toxicity of contaminated feeds.

### 3.7. Antioxidant Mixture

Melatonin (Mel) and coenzyme Q_10_ (CoQ_10_) are well-known antioxidants and free radical scavengers. Mel, N-acetyl-5- methoxytryptamine, is a hormonal product of the pineal gland that plays many roles within the body, including control of reproductive functions, modulation of immune system activity, limitation of tumorigenesis and effective inhibition of oxidative stress [164]. CoQ_10_ is an integral component of the mitochondrial oxidative phosphorylation system and is a lipid-soluble redox carrier between particular respiratory enzyme complexes in the electron transport chain in the mitochondrial inner membrane [165]. Yenilmez *et al*. investigated the effects of a relatively high single-dose of OTA and the antioxidant effects of Mel and CoQ_10_ on OTA-induced oxidative damage in rats [166]. Male Sprague-Dawley rats were divided into four groups of seven rats each: control, OTA, Mel + OTA and CoQ_10_ + OTA groups. Malondialdehyde (MDA) levels in the plasma and GSH levels in whole blood were measured; kidneys (for histological inspection and for apoptosis detection by TUNEL method) and bone marrow samples (for chromosome aberration and mitotic index) were taken. The rats in the OTA group showed limited degeneration of tubular cells. In some tubules, karyomegaly, desquamated cells and vacuolization were observed by light microscopy. Mel and CoQ_10_ treatment significantly reduced the severity of the lesions. The MDA levels of the OTA group were significantly higher than the control, OTA + Mel and OTA + CoQ_10_ groups, while GSH levels were significantly lower than the control, OTA + Mel and OTA + CoQ_10_ groups. Higher incidences of apoptotic bodies were observed in the kidneys of the OTA group, although OTA administration did not significantly change the incidence of apoptotic bodies when compared to the control and antioxidant-administered groups. Although the percentage of the mitotic index was lowest in the OTA group, no statistical difference was found among the groups. Additionally, OTA had no numerical and structurally significant effects on chromosomes. It was observed that single-dose OTA administration caused oxidative damage in rat kidney, and Mel or CoQ_10_ treatment appeared to ameliorate the OTA-induced tissue injuries. Atroshi *et al*. performed a study of the appearance of liver apoptosis after OTA administration in male mice [167]. They demonstrated that the administration of OTA twice a week for one- or two-week periods resulted in the occurrence of apoptosis in mice liver. The presence of intracellular apoptosis bodies was detected at two weeks after toxin treatment. Light microscopic examination demonstrated the presence of eosinophilic globules, often containing apoptotic bodies. Moreover, the ability of selenium combined with other antioxidants, such as CoQ_10_, L-carnitine, Zn, Mg, N-acetyl cysteine, vitamin C, vitamin E or tamoxifen, to intervene in apoptosis induced by OTA in livers of mice was also investigated. The authors demonstrated that the antioxidants have inhibitory effects on OTA-induced apoptosis. The cellular redox state and/or the equilibrium between ROS generated by OTA and ROS detoxification by the antioxidants could influence the early stage of apoptosis.

## 4. Conclusions

In conclusion, several mechanisms have been proposed for OTA toxicity and OTA renal tumor formation: inhibition of protein synthesis, interference with metabolic systems, promotion of membrane lipid peroxidation, disruption of calcium homeostasis, inhibition of mitochondrial respiration and DNA damage. A growing number of *in vitro* and *in vivo* studies has been collected and describe evidence compatible with a role for oxidative stress in OTA toxicity and carcinogenicity. For these reasons, several studies have been performed using antioxidants to try to counteract the adverse effects of oxygen radicals generated under OTA-treatment.

These studies demonstrated that antioxidants are able to counteract the deleterious effects of chronic consumption of OTA and confirmed the potential effectiveness of dietary strategies to counteract OTA toxicity.
